# Fabrication and characterization of metformin-loaded PLGA/Collagen nanofibers for modulation of macrophage polarization for tissue engineering and regenerative medicine

**DOI:** 10.1186/s12896-023-00825-2

**Published:** 2023-12-19

**Authors:** Akram Firouzi Amandi, Seyed Abbas Shahrtash, Shaylan Kalavi, Afshin Moliani, Hanieh Mousazadeh, Mehdi Rezai Seghin Sara, Mehdi Dadashpour

**Affiliations:** 1 Student Research Committee, Tabriz University of Medical Sciences, Tabriz, Iran; 2 Department of Pharmaceutical Engineering, University of Tehran, Tehran, Iran; 3 Department of Clinical Pharmacy, Faculty of Pharmacy, Islamic Azad University of Medical Sciences, Tehran, Iran; 4 Isfahan Medical Students Research Center, Isfahan University of Medical Sciences, Isfahan, Iran; 5 Research Institute of Bioscience and Biotechnology, University of Tabriz, Tabriz, Iran; 6 Stem Cell Research Center, Tabriz University of Medical Sciences, Tabriz, Iran; 7 Department of Medical Biotechnology, Faculty of Medicine, Semnan University of Medical Sciences, Semnan, Iran

**Keywords:** Metformin, Electrospun nanofiber, Macrophage repolarization, Regenerative medicine

## Abstract

In tissue engineering (TE) and regenerative medicine, the accessibility of engineered scaffolds that modulate inflammatory states is extremely necessary. The aim of the current work was to assess the efficacy of metformin (MET) incorporated in PLGA/Collagen nanofibers (Met-PLGA/Col NFs) to modulate RAW264.7 macrophage phenotype from pro-inflammatory status (M1) to anti-inflammatory status (M2). Given this, MET-PLGA/Col NFs were fabricated using an electrospinning technique. Structural characterization such as morphology, chemical and mechanical properties, and drug discharge pattern were assessed. MTT assay test exposed that MET-PLGA/Col NFs remarkably had increased cell survival in comparison with pure PLGA/Collagen NFs and control (*p* < 0.05) 72 h after incubation. Based on the qPCR assay, a reduction in the expression of iNOS-2 and SOCS3 was found in the cells seeded on MET-PLGA/Col NFs, demonstrating the substantial modulation of the M1 phenotype to the M2 phenotype. Moreover, it was determined a main decrease in the pro-inflammatory cytokines and mediator’s expression but the growth factors amount related to anti-inflammatory M2 were meaningfully upregulated. Finally, MET-PLGA/Col NFs possibly will ensure a beneficial potential for effective variation of the macrophage response from an inflammatory phase (M1) to a pro-regenerative (M2) phase.

## Introduction

The most common wounds in the world, after surgical incisions and slight abrasions, are diabetic, venous ulcers, and pressure sores [1]. While surgical incisions and lacerations are classified as a critical wounds that heal quickly, wounds with little sequelae are chronic wounds that do not heal and demand effective treatments [2]. Recently, there have been 4.5, 9.7, and 10 million cases of pressure ulcers, venous ulcers, and diabetic ulcers, respectively [1]. Regrettably, the soaring incidence of chronic, non-recovery wounds is outpacing the development of new, effective treatments [2]. Macrophages are highly elastic and adaptive cells that may be maintained in a variety of pro-inflammatory phenotype (M1) or anti-inflammatory phenotype (M2) as a result of environmental signals [3]. Numerous studies have shown that macrophages serve multiple roles as regulators of wound healing, and that they play several functions to guarantee adequate healing [2]. It is widely known that the phenotype of macrophages changes as wound healing progresses [4]. After an injury, pro-inflammatory macrophages, also known as “M1” macrophages, invade the site to clear it of germs, foreign debris, and dead cells [5]. To ensure successful microbial eradication, these macrophages secrete inflammatory cytokines including IL-6, IL-1β, and TNFα, as well as increase the production of reactive oxygen and nitrogen species and increase the expression levels of MHC I/II, CD80, and CD86 [6]. In acute wounds, the general population of macrophages changes to a population with anti-inflammatory effects (called M2 macrophages), as well as the proliferation and migration of endothelial cells, keratinocytes and fibroblasts to repair the vasculature, epidermis, and dermis in that order, when the tissue begins to repair [5]. Anti-inflammatory cytokines such TGF-β, IL-4, and IL-10 are abundant in M2 macrophages [5]. The anti-inflammatory and regenerative abilities of M2 macrophages are also associated with the enhanced effect of Arg 1 and the mediated surface expression of the macrophage mannose receptor (MMR) [7]. Macrophages also play a critical function in vascularization, assisting in the stability and fusing of newly formed blood vessels by placing themselves near them [8]. Therefore, regulating the phenotypic switch of macrophages from an inflammation (M1) to proliferative process (M2) at specific interval may offer a principal viable technique in regenerative medicine [9].

Several natural and synthetic drugs have been found to control the inflammatory pathway from M1 to M2 [10]. Recently, several studies have effectively demonstrated that the inflammatory pathway may be switched from an inflammatory pathway to an anti-inflammatory pathway by the pharmacological application of several natural analogues [11]. MET is a synthetic guanidine derivative derived from Galega officinalis extracts [12–14]. Although, MET is one of the blood sugar-lowering drugs often prescribed for people with type 2 diabetes, little research has been done on its effect on wound healing [15]. MET suppressed the generation of pro-inflammatory cytokines, prevented oxidative damage, and directed macrophage polarization in vivo and in vitro, according to new findings [16]. Medicinal nanoencapsulation is gaining traction as a viable option for reducing drug adverse effects while achieving controlled release and site-specific delivery [17, 18]. MET-loaded nanocarriers have been rationally engineered in recent years to increase their therapeutic effectiveness in anti-inflammatory, weight-loss, and neuroprotection [19].

Polymers have long been touted as the most alternative for encapsulating a wide range of molecules, such as pharmaceuticals. In addition, they are biocompatible and biodegradable, as well as release desired load through several mechanisms, including temperature, pH, conjugation, and surface modification [20, 21]. Polylactic-co-glycolic acid (PLGA) is a copolymer synthesised from glycolic acid and lactic acid used for controlled drug delivery of small molecules approved by the Food and Drug. Administration (FDA) [22]. It is now widely used in biomedical engineering, mainly for the controlled release of drugs, bone materials and surgical suture materials for eye surgery [23]. PLGA can easily be formed into designed shapes with relatively high mechanical strength, but their hydrophobic surface is not favorable for cell seeding [24]. Collagen is the main structural protein in the extracellular matrix and collagen fibers are capable of accelerating wound healing process [25, 26]. Since Collagen is highly hydrophilic, it can improve the interaction of cells with the scaffold. Also, it has the ability to trigger biological signals to support cell adhesion and proliferation [27]. In current years, blends of collagen with other polymers have been investigated for development of new materials. Such materials may show improved characteristics required in the biomedical field and cosmetic preparations [28].

Incorporated of MET in polymeric nanofibers can give a prolonged release and greater effectiveness at lower doses. Furthermore, adverse effects can be controlled by using a lower dose form [29]. So far, several approaches have been discovered to generate fibers, such as electrospinning, that involves attractive/repulsive forces to form fiber polymeric structures of different sizes [30]. Electrospinning is easy and useful process for modifying and regulating the shape, diameter, and surface characteristics of fibers [31, 32].

**Fig. 1:**
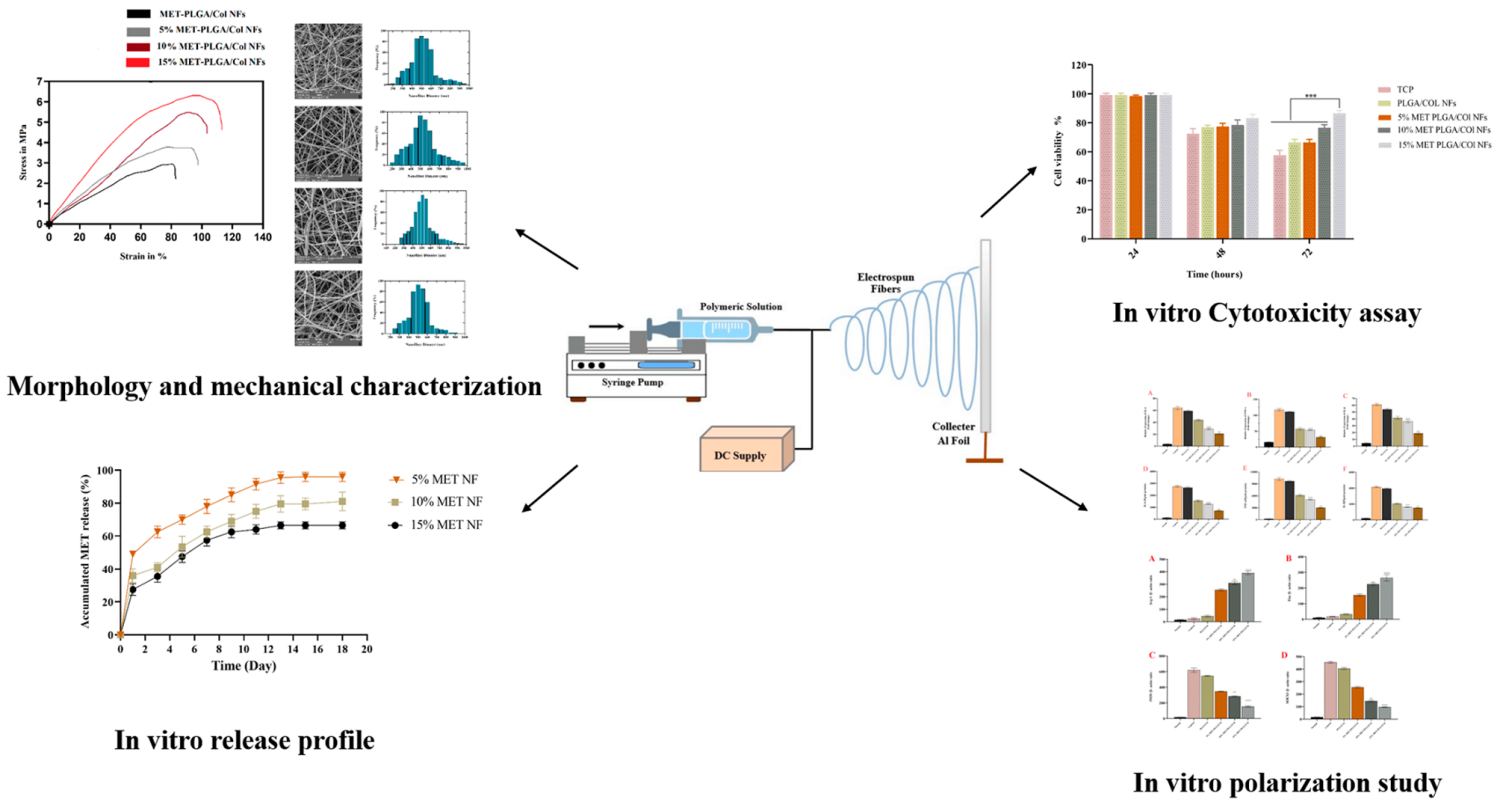
Schematic illustration of the present study

Herein, we hypothesized that combining MET, a natural immunomodulatory drug, with polymeric nanofibers might provide a unique platform to deliver MET sustainably to repair infected and diabetic wound. As a result, we incorporated MET into PLGA-Col NFs and tested their ability to shift macrophage functional polarity toward anti-inflammatory M2 phenotypes. Furthermore, morphological assessment, chemical and physical composition, drug release behaviors, and *in-vitro* cytotoxicity assessment of NFs were studied. An overview of our work is shown in Fig. 1.

## Materials and methods

Polylactic-co-glycolic acid (PLGA) (PLA, Mw ~ 160,000 g/mol), Metformin hydrochloride (Merck, ≥ 99.9%), Dimethyl sulphoxide (DMSO), MTT powder (3(4, 5-dimethyl thiazol-2-yl) 2, 5-diphenyl-tetrazolium bromide), Collagen type I, and 1,1,1,3,3,3-hexafluoro-2-propanol (HFIP) were purchased from Sigma–Aldrich (Saint Louis, MO, USA). Fetal bovine serum (FBS), Dulbecco’s Modified Eagle’s Medium (DMEM), pen-strep, trypsin EDTA, and TRIZol reagent were obtained from Gibco (Invitrogen, Carlsbad, CA).

### Methods

#### PLGA-Col NFs fabrication

PLGA and collagen with a ratio of PLGA: Col 4:1 were dis solved in HFIP as common solvent (20% w/v). The reaction was then followed under magnetic stirring condition at room temperature for 16 h. Also, the resultant was irradiated in an ultrasonic bath for another 2 h to remove air particles that were formed during the fusion process. Finally, the polymer solution was then injected into a high-voltage electrostatic spinner machine in a plastic syringe (2.5 mL, 500 mm, TL-01, Shenzhen, China). With a mixture input rate of 1.2 ml/h, the electrospinning method was performed under a high voltage power source of 15 kV. A rotating ground cylinder measuring 20 × 15 cm was employed 12 cm from the needle to collect the electrospun membranes. Aligned parallel fibers were stacked at a rotation speed of 1000 revolutions per minute (RPM), while random fibers were stacked at 10 rpm [33]. The PLGA/Col NFs obtained from electrospinning were dried in a vacuum-drying oven for 48 h to remove the residual organic solvent.

#### Fabrication of MET-loaded PLGA/Col NFs

To synthesize MET-loaded PLGA/Col, an appropriate amount of MET (5, 10 and 15 wt %) with respect to the PLGA/Col content was added to PLGA and Col solution with a weight ratio of 80:20 (wt:wt%) and stirred magnetically overnight at 25 °C. The electrospinning condition to generate electrospun nanofibers was optimized as followed: voltage in the range of 20 kV, collecting distance of 18 cm, a flow rate of 0.3 mL.h-1, and ambient conditions of 40 °C. The mats were dried in a vacuum dryer for 48 h in a vacuum-drying oven to remove the residual solvent, and this sample was applied for further characterizations.

#### Evaluation of drug loading percentage

The drug-loaded NFs membranes were cut into a square sample of 5 × 5 cm, and the mass was precisely calculated and dissolved DMSO of 5 mL. After complete dissolution, 0.5 mL was taken and diluted to 10 mL. The amount of the MET was detected at 280 nm with an UV–Vis spectrophotometer, in which the concentration was obtained using a standard curve from known concentrations of MET solutions.

#### Characterization of MET-loaded PLGA/Col NFs

##### Scanning electron microscopy analysis

The pore morphology, size, distribution, and interconnectivity of the electrospun NFs was characterized using field emission scanning electron microscopy (FE-SEM) (MIRA3 TESCAN, Czech) at 25 kV. The average diameter and the distribution of the NFs were determined from the FE-SEM photographs using image analysis software (Image J, National Institutes of Health, Bethesda, VA).

##### Fourier-transform infrared (FTIR) analysis

To identify the structural information and chemical analysis of samples was determined by Bruker Tensor 270 spectrometer spectroscopy over a range of 4000–400 cm^− 1^ [34, 35].

##### Mechanical properties

The mechanical properties of the electrospun fibrous membranes were determined using a material testing machine (Shimadzu; Kyoto Japan). The tensile specimens were prepared by fixing the nanofiber on a stretching fixture, which the gage length L was 15 mm and the crosshead speed was 5 mm/min. All samples were prepared in the form of a rectangular shape with dimensions of width × length = 4 mm × 20 mm from the electrospun fibrous membranes [36].

#### Drug release study

MET was released from PLGA/Col NFs at 37 °C. In a centrifuge tube (15 mL), PBS (pH 7.4, 10 ml) was included to an electrospun PLGA/Col fibrous film (area: 3 cm^2^) that had been combined with MET. At predetermined time points, 3 mL of the buffer was replaced with by another 3 mL of fresh PBS (pH 7.4) to maintain a constant volume. Utilizing UV-Vis spectrophotometry (PerkinElmer Fremont, CA), [37] the absorption at 280 nm was tested to assess the consistency of MET in the release solution. Then, it was calculated how much MET was released as a function of time. The measures were all done three times.

#### Isolation and purification of C57BL/6 mouse macrophages

All of the in vivo trials were approved using the Guideline for the Care and Use of Laboratory (The Ministry of Health and Medical Education of Iran) adjusted according to the National Research Council’s Guide for the Care and Use of Laboratory Animals (IR.TBZMED.VCR.REC.1400.071) [38]. Mice of the C57BL/6 strain, aged 6–8 weeks, were bought from the Pasteur Institute’s Animal Breeding Facility Centre (ABFC) in Karaj, Iran. Tibias and Femurs were taken out in bilaterally to acquire bone marrow-derived macrophages (BMDMs), and marrow core were washed utilizing DMEM containing FBS. Then, mice were euthanized with 100 µL of a 10:1 mixture of ketamine (100 mg/mL; Ketaset, Fort Dodge, Fort Dodge, IA) and xylazine (100 mg/mL; Anased, Lloyd Laboratories, Shenandoah, IA) delivered either retroorbitally or by injection into the lateral tail vein. After a medium wash, cells were cultured in DMEM enriched with FBS (10%), 1% penicillin/streptomycin, and 10 ng/ml recombinant mouse CSF (PEPROTECH #315 02). M CSF induces a macrophage phenotype in bone marrow cells (7–10 days). According to F4/80 immunohistochemical staining, macrophages were present in 99 ± 0.8% of cells. After digestion, cells were resuspended in macrophage medium.

#### Cell viability assay

Phosphate buffered saline (PBS) was used to wash the samples three times. Then, BMDMs were exposed for one week with fibers containing MET at various concentrations. The prepared samples were then incubated using CCK-8 reagent (Biotim, China) at a proportion of 1:10 (CCK 8: medium). Cells were kept at 37 °C for 2 h. A 100 mL aliquot was then added to a 96-well plate and the absorbance was read utilizing an ELISA reader at 450 nm (Multiskan MK3, Thermo Scientific, USA).

#### Gene expression analysis

By monitoring changes in the gene expression marker of M1 (iNOS2) and M2 (Arg1) expression, and inflammatory mediators such as IL-1β, IL 6 and TNF-α in BMDMs, it was possible to assess the extent of well-being. MET-loaded PLGA/Col NFs functioned in vitro to regulate the polarization of macrophages from pro-inflammatory to anti-inflammatory subtype and their M2 phenotype properties. By seeding macrophages induced with LPS on electrospun NFs (1 cm^2^) in 24-well plates, Real Time PCR test was evaluated. After 72 h, RNA was separated using of the TRIzol reagent based on the manufacturer’s instructions. quantity and quality of RNA were measured by the NanoDrop spectrophotometer (ND-2000, Thermo Fisher Scientific, Waltham, MA). In addition, the isolated RNA integrity was evaluated by gel electrophoresis (1.5% agarose gel) containing GelRedTM (Biotium, Inc., Hayward, CA). Based on the manufacturer’s recommendation SYBR Premix EX Taq II for Qpcr, the PrimeScript kit (Takara, Dalian, China) was applied to generate cDNA from total RNA (500 ng). Then, the test was carried out utilizing a combination of primers, SYBR Premix EX Taq II, cDNA, and deionized water. A reaction cycle at 95 °C for 5 min was followed by 38 cycles of decomposition (96 °C for 12 s), heating (54 °C for 30 s), and extension (74 °C for 18 s). Lastly, melting curve analysis was performed at 65–95 °C using the amplicon as a result, β-actin gene as a homemaking gene was utilized in qPCR to measure gene expression level using the ΔΔCt method. For each sample, three replicates were performed.

#### Cytokine release from cells

Production of cytokines was measured from prompted macrophage seeded (10^6^) on electrospun NFs (1 cm^2^ / 24 well). 2 hours later, the supernatant was collected to perform an ELISA test to quantify the levels of M2 phenotype-related growth factors bFGF, VEGF, and TGF-β1, also pro-inflammatory cytokines comprising IL-6, IL-1β and TNF-α. All ELISA kits have been bought from eBioscience Affymetrix Company.

#### Statistical analysis

Three independent experiments were performed for each trial. The outcomes were stated using mean and standard deviation. All statistical analysis was carried out with GraphPad Prism (version 6.01). A two-way ANOVA test was applied to compare the results. (Statistical importance is shown by *P* < 0.05).

## Results and discussion

### NFs characterization

The electrospinning-fabricated fiber membrane has high porosity, hole interconnectivity, and specific surface for cell adhesion [39, 40]. The capacity of electrospun fibers to resemble the extracellular matrix (ECM) helps to reduce wound progress by increasing hemostasis and wound secretions absorption [41]. Electrospun nanofibers (NFs) have been discovered that have wide applications in wound healing filtration [42], protective clothing [43], drug delivery [44], and medicinal fields [45] such as scaffolding for tissue engineering [46]. Biodegradable materials have been broadly used in medical treatment due to their probable degradation products, suitable biocompatibility, and identical degradation mechanisms in vitro and in vivo [47]. In the current study, two FDA approved and broadly used biopolymers for the construction of tissue engineering scaffolds, PLGA, and Collagen were chosen [48]. PLGA is a biocompatible and biodegradable linear co-polymer and has been approved by the FDA for antigen delivery.

**Fig. 2:**
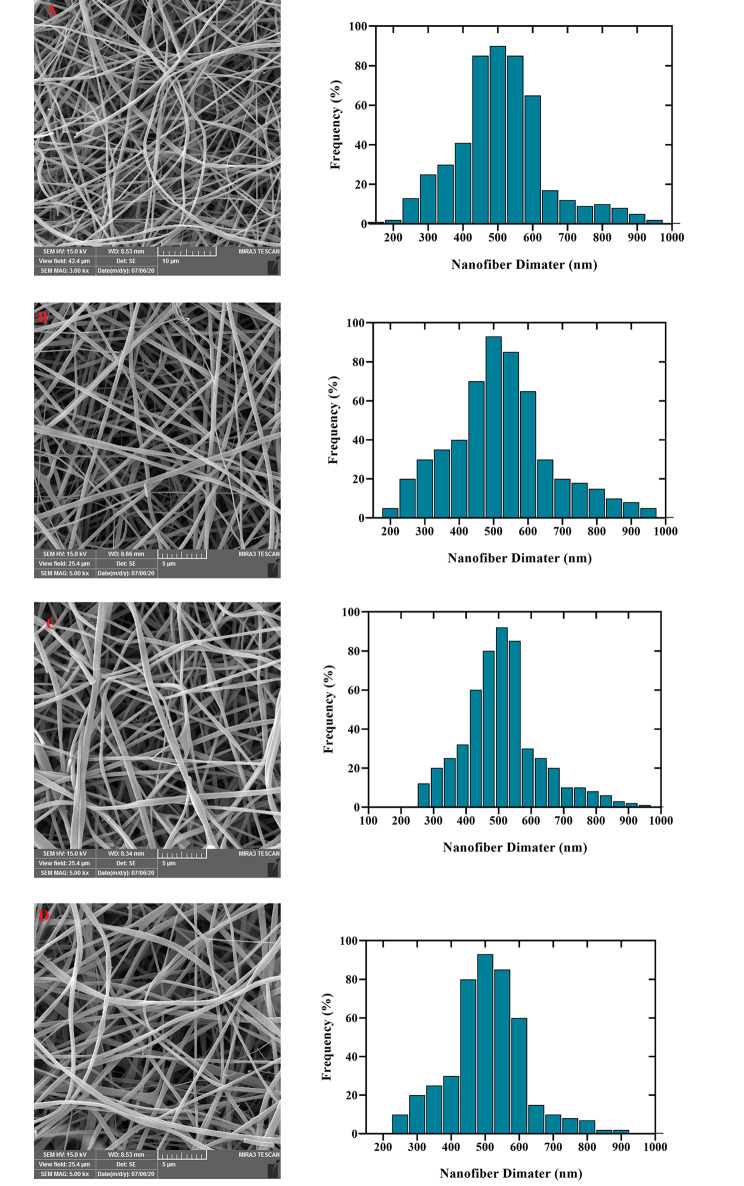
FE-SEM images and corresponding diameter distribution for PLGA (**A**), PLGA/Col (**B**), 5% MET- PLGA/Col (**C**) and 15% PLGA/Col (**D**) electrospun fibers, respectively

The FE-SEM micrographs of electrospun PLGA/Col NFs and MET-PLGA/Col NFs are shown in Fig. 2. These randomly oriented nanofibers were found to have a smooth, bead-free surface and nearly consistent diameters throughout their lengths. The diameter distribution of the NFs was not affected by MET loading. These composite nanofibers’ sizes were found to be between 150 and 300 nm (Fig. 2C). In comparison to NFs loaded with only PLGA/Col, the presence of a 20 wt% significantly of MET did not considerably change the shape or frequency of fiber dispersion. Additionally, the surface of the fibers did not contain any MET aggregates. Under optimal conditions, a 20% (w/w) maximum concentration of MET could be loaded into PLGA/Col NFs to produce bead-free electrospun meshes. The fibers in the PLGA/Col electrospun mat revealed modest connectivity between them, but the hybrid scaffolds including MET displayed a point-bonded fiber structure (Fig. 2 (A, B)).

Because of biocompatible, biodegradable, non-toxic, and non-immunogenic qualities of PLGA-Col-based NFs, they have been proposed as promising nano-delivery method [49, 50]. Samy and et al. fabricated poly(ε-caprolactone)/gelatin (Gel) nanofiber mats (5-FU-PCL/Gel NFs) and showed that the loading of 5-FU with three different concentrations (5, 10, and 15 wt%) enhanced PCL stabilization in the formic acid/acetic acid system [51]. In another study, PLGA/GEL NFs sheet materials loaded with mesoporous silica nanoparticles were manufactured using an electrospinning technique. The average diameters of the NFs were 641 ± 24 nm and 418 ± 85 for pure PLGA NFs and PLGA scaffolds/silica nanoparticles respectively [52]. Ranjbar-Mohammadi and et al. successfully fabricated curcumin (Cur)-loaded NFs and showed that enhance of Cur concentration resulting in the increase of PCL fiber diameter, narrowing the diameter distribution, and to the increase of polymer solution viscosity [53].

### Mechanical properties

**Fig. 3:**
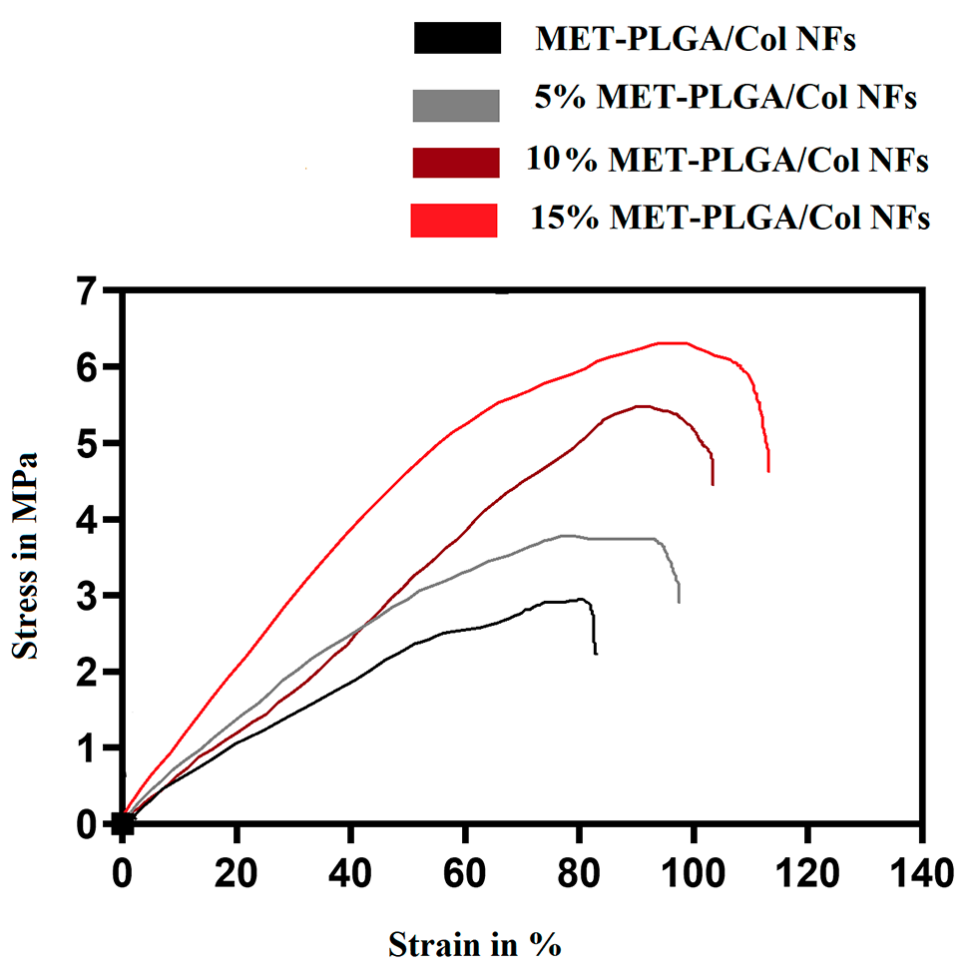
Tensile stress–strain curves for PLGA/Col fiber mats spun with different additive content

Mechanical properties hold dominant significance for nanofibrous scaffold, as the underlying matrix must have strong mechanical strength to effectually ease tissue repair [54]. Assessing the nanofibrous scaffolds’ tensile strength (TS) calculated in MPa was completed through the assessment of strain-stress curves. Figure 3 shows the stress strain curves of PLGA/Col and 5% MET-PLGA/Col NFs, 10% MET-PLGA/Col NFs and 15% MET-PLGA/Col NFs mats. The 5% MET-PLGA/Col NFs mat displayed mechanical properties with a TS of 3.5 MPa and elongation at break of 97%. The nonwoven mat of 15% MET-PLGA/Col NFs had a TS of 6.2 MPa and elongation at break of 119%. We found that the TS of MET-PLGA/Col NFs were greater than PLGA/Col NFs. As displayed in Fig. 3, TS of blended mat was enhanced with the presence of MET. The nanofibrous mats need a specific level of mechanical strength since they would be utilized as an effective scaffold in TE.

### FTIR

**Fig. 4:**
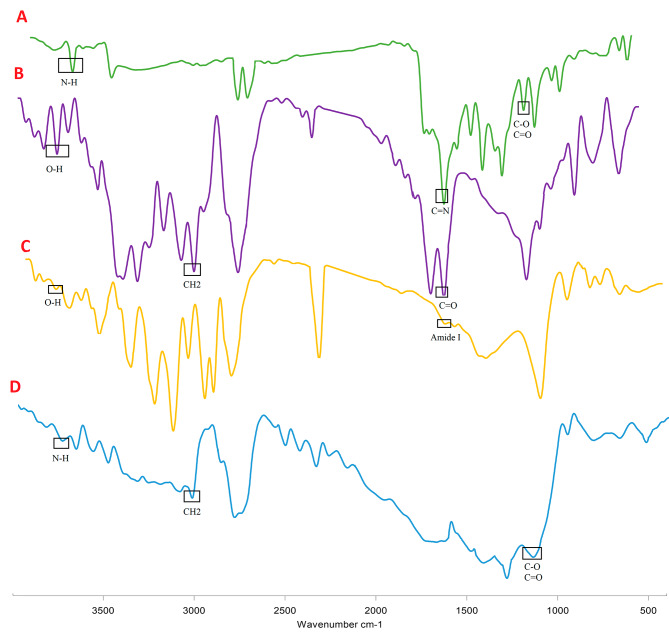
FTIR results of MET (**A**), PLGA (**B**), PLGA/Col (**C**) and MET- PLGA/Col NFs (**D**)

FTIR was used to investigate the interactions between MET and PLGA/Col functional groups (Fig. 4). The main PLGA typical bands at 2942 and 2866 cm^− 1^ attributed to the asymmetric and symmetric stretching of CH_2_ bonds, respectively. Also, the distinctive peaks pertaining to PLGA at 1720 cm^− 1^, and 1240 cm^− 1^ were assigned to the C = O, C-O stretching vibrations. The typical peaks of amide I and amide II (N-H Transformation) for Col fibers were found at 1651 and 1540 cm^− 1^, respectively. In fibrous mats, new peaks were created by the addition of MET. It is found that C-H is known bands at 2631, 2710 and 2921 cm^− 1^. In addition, a double band can be seen in the ɣ benzopyrone ring in the region of 1653 cm^− 1^ corresponding to the stretching vibrations of carbon in the benzene and ɣ pyrone rings, as well as the absorption bands in the regions of 1612, 1576 and 1450 cm^− 1^ attributed to the vibrations of the C–O group. The findings showed the association and existence of MET in PLGA/Col NFs. Drug-polymer systems may interact through hydrogen bonds leading to systems with much-improved drug loading, dissolution performance, and overall stability [55].

### MET loading percentage and release from MET-PLGA/Col NFs

**Fig. 5:**
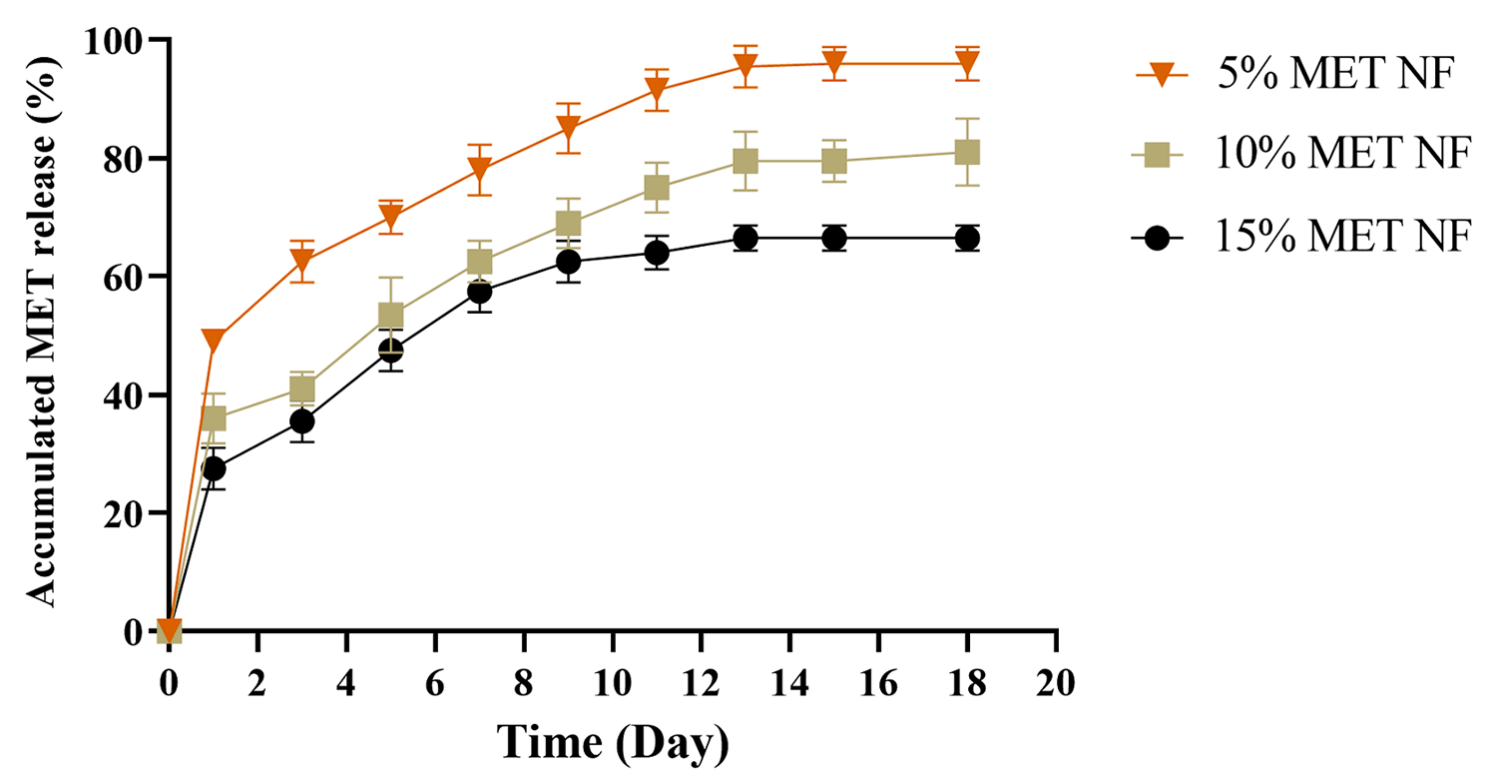
Cumulative release profiles of MET from 5% MET- PLGA/Col NFs and 15% MET- PLGA/Col NFs - PLGA/Col electrospun fibers. Results are expressed as the mean ± SEM (n = 3)

Therapeutic biomaterials release pattern from the carrier-system has a high correlation with the therapeutic efficacy [56]. The outcomes presented that 83% of the applied drug was loaded in the NFs. Figure 5 shows the results of this investigation of the MET release profile of electrospun PLGA/Col NFs as a function of immersion time. A burst release of MET was significant for the initial 24 h in a human physiology simulation (37 °C, pH = 7.4), and it was followed gradually released over the next 2 weeks. Approximately 92% of MET were released from MET-loaded fibers after 2 weeks. The initial burst release might had been caused by rapid solubilization of amorphous drug-aggregate on the nanofiber surface and leaching of near-surface entrapped drugs in direct contact phosphate buffer. The primary explanation of the sustained release is a longer diffusion path from the thick nanofibers, as well as slow degradation of the PLGA/Col fibers [57]. In a study, glybenclamide(Gb) and MET-loaded bacterial cellulose/gelatin NFs synthesized and evaluated drug release behaviors and *in-vitro* cytotoxicity. The results reveal that Met and Gb loaded NFs in a sustained release form has high potential for diabetic wound healing with high bioavailability and fewer systemic side effects [58]. A previous study developed a biodegradable core-shell NFs loaded with fish sarcoplasmic protein (FSP) and MET to investigate drug release profile for diabetic wound healing uses. They result showed that the prepared NFs with prolonged and sustained release characteristics had a good potential to be used in the treatment of diabetic ulcers [59].

### Macrophage viability

**Fig. 6:**
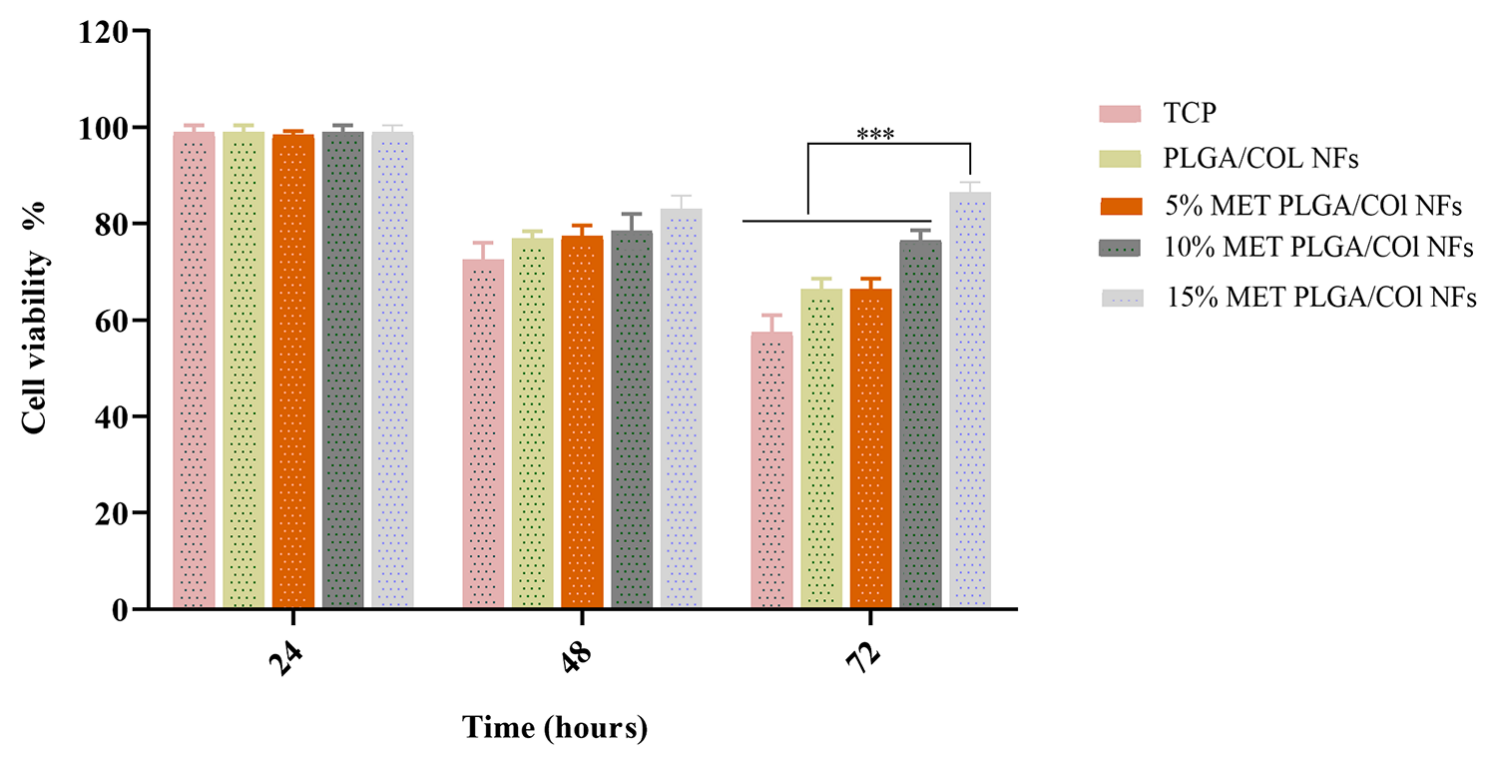
In vitro Cytotoxicity of MET-PLGA/Col NFs on LPS-induced RAW264.7 macrophage cells assessed after 3 days’ incubation using MTT colorimetric assay (*P* < 0.05). Values are expressed as mean ± SD of three parallel measurements

Electrospun nanofibrous scaffolds must assess biocompatibility and cytotoxicity tests before being used in regenerative medicine and tissue engineering [60]. At 72 h after cell seeding on TCP, PLGA/Col NFs, 5% MET-PLGA/Col NFs, 10% MET-PLGA/Col and NFs 15% MET-PLGA/Col NFs, cell viability was evaluated using the CCK-8 colorimetric assay to investigate the potential cytotoxicity of MET-PLGA/Col NFs on stimulated macrophage cells. As a displayed in Fig. 6, the presence of MET into PLGA/Col NFs effected improved cell viability after 3 days of incubation, proposing that cellular uptake as well as intracellular release of MET from NFs take place relatively slow without affecting viability of the cells.

Though, viability of macrophages on PLGA/Col NFs and MET-PLGA/Col NFs were like after 1 d of culture, substantially higher cell viability was detected on MET-PLGA/Col NFs in comparison with PLGA/Col NFs after culture for 3 d. These outcomes prove that MET-PLGA/Col NFs have high biocompatibility, minimal toxicity and biomimetic features in comparison with the PLGA/Col NFs. Furthermore, using co-polymerized PLGA with Col has prepared highly biocompatible, nonimmunogenic, biodegradable, hydrophilic, and non-toxic biopolymer [48]. These results were similar with the preceding studies showed by our team and in which it was discovered that Chrysin-loaded PLGA/PEG nanoparticles in comparison with free Chrysin have an insignificant effect on the proliferation and viability of the macrophages [10].

These results demonstrate that EO-PCL/PEG nanofibers have high biocompatibility compared with PCL/PEG nanofibres.

### In vitro repolarization study

**Fig. 7:**
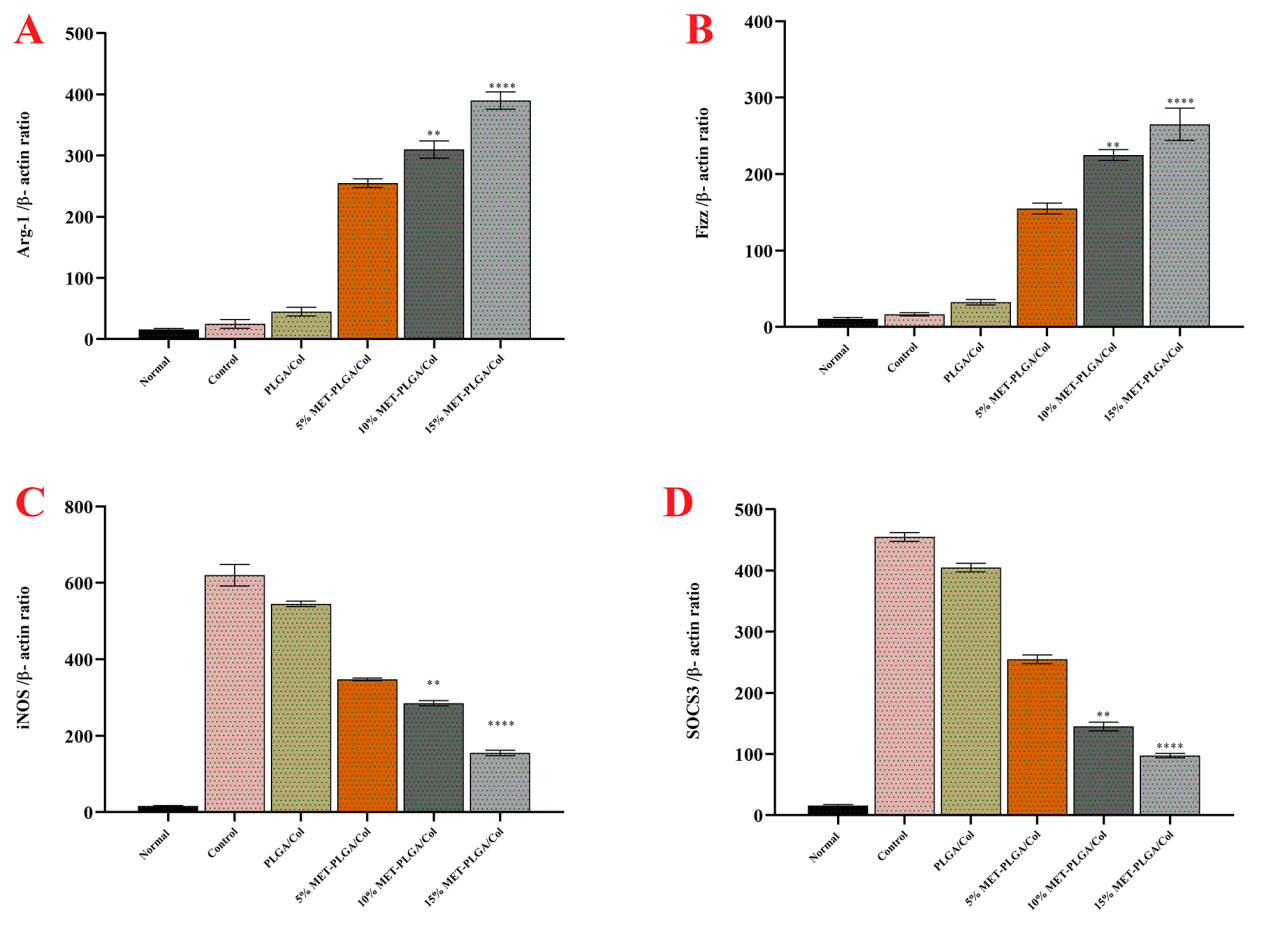
In vitro polarization study. Relative mRNA expression levels of M2 markers (**A**) Arg-1 and (**B**) Fizz and M1 markers iNOS (**C**) and SOCS3 (**D**) in macrophages treated with MET-PLGA/Col NFs in various concentrations after 24 h incubation. (*P < 0.05, **P < 0.01; ***P < 0.001), Results are mean ± SEM (n = 3)

qPCR was used to measure the expression of iNOS, a marker of M1 macrophages, and Arg-1, a marker of M2 macrophages, in LPS/IFN γ-stimulated bone marrow-derived macrophages grown on NFs for 72 h to investigate its efficacy. MET-PLGA/Col NFs for macrophage response from inflammation (M1) to regeneration phase (M2) (Fig. 7).

Arginase-1, an enzyme that prevents the formation of extracellular NO by metabolizing its precursor L-arginine to ornithine and polyamines, is produced by M2 macrophages [61].

First, it was discovered that LPS/IFN-γ treated cells in culture expressed more iNOS than untreated cells, this shows that the cell’s polarization towards the inflammation phenotype (M1) was effective, that the electrospun nanofibrous scaffolds were able to increase the arg1 expression in LPS/IFN γ induced cells 72 h after incubation, but the scaffolds without met had a significant effect. Moreover, iNOS production in LPS/IFN γ-stimulated cells significantly decreased with MET-PLGA/Col NFs after three days of culture, but iNOS expression level was maintained in cells cultured in NFs without MET. These results indicated that the repolarization of bone marrow-derived macrophages from the M1 to the M2 phenotype may be successfully facilitated by MET containing electrospun nanofibrous scaffolds. The expression profile of certain biomarkers, such as chemokines, cytokines, and surface markers secreted in response to regional environmental stimulus, is used to define the phenotype of macrophages. The known M1 phenotypic marker iNOS is controlled by a range of lipid mediators and inflammatory cytokines Overproduction of nitric oxide (NO) is activated by nuclear factor kappa B (NF-κB) signaling in response to inflammatory stimuli [62]. On the other hand, M2 phenotypes were discovered to show higher amounts of Arg1 and lower amounts of iNOS. The iNOS/Arg1 proportion is often used to explain a simple method to determine whether a polarized macrophage is M1 or M2 [63]. In a study, anti-inflammatory potential effect of Phyllolobium chinense Fisch flavonoids (PCFF) was assessed in *in vivo and in vitro*. Their results showed that PCFF meaningfully reduced NO overproduction and iNOS gene overexpression in LPS sitimulated pro-inflammatory M1 macrophages [62]. Farajzadeh and et al. synthetized Cur loaded-CD44-targeting HA-PLA NPs and evaluated the effect of free and nanofurmolated of Cur on the modulation of macrophage polarity. They proved that Cur loaded-CD44-targeting HA-PLA NPs reduced inflammation in peritoneal macrophages affected by LPS by reducing of pro-inflammatory expression level [5].

**Fig. 8:**
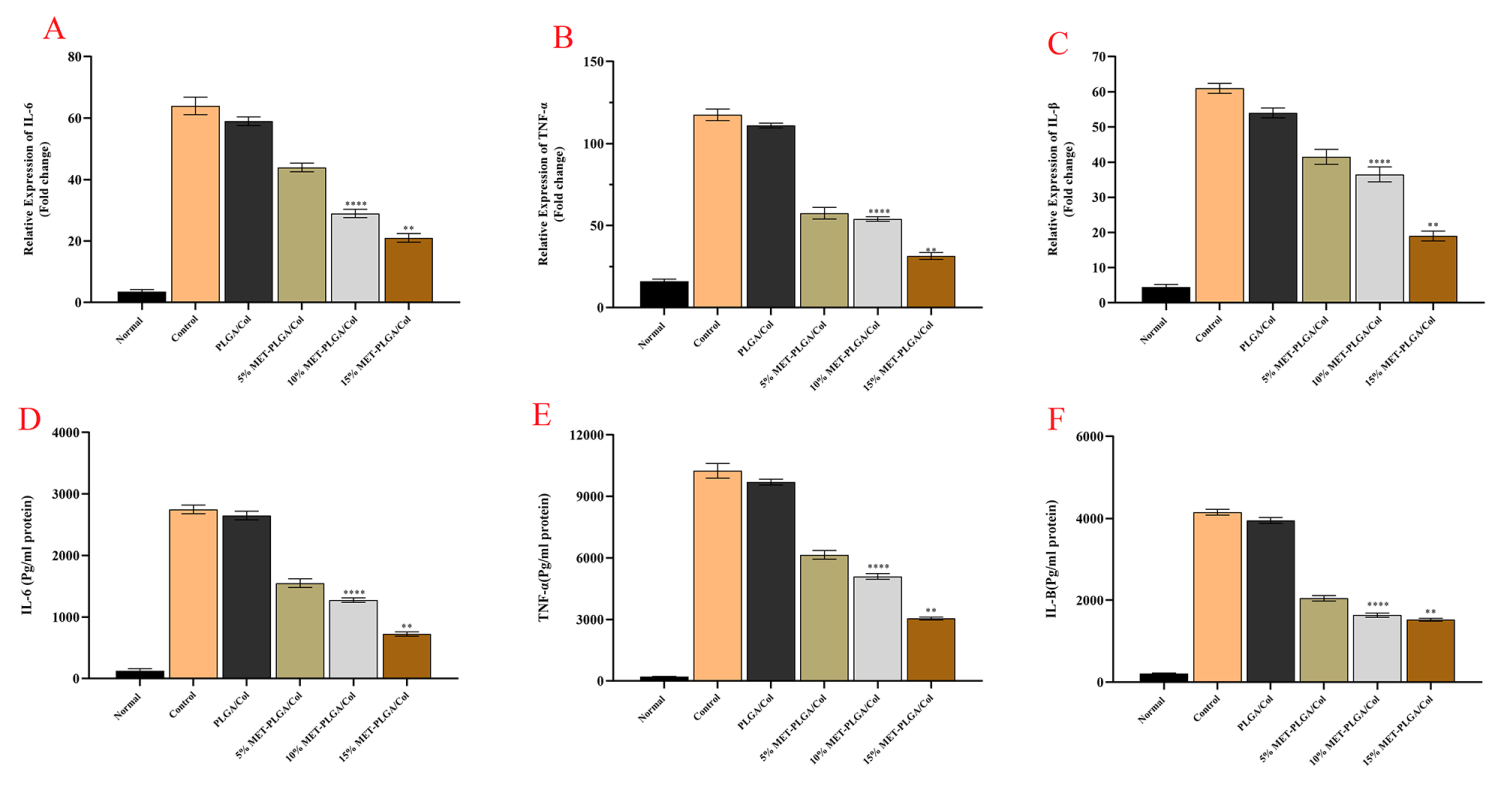
The anti-inflammatory effect of MET-loaded PCL/GEL NFs evaluated on RAW264.7 cells stimulated with LPS/INF-γ. Histograms show the mean values of protein levels of (**A**) IL-6, (**B**) TNF-α, and (**C**) IL-1β and the amounts of (**D**) IL-6, (**E**) TNF-α, and (**F**) IL-1β after ELISA evaluation. All values are expressed as mean ± SEM. *P < 0.05, **P < 0.01; ***P < 0.001

In vitro anti-inflammatory properties of MET-PLGA/Col NFs on the induction (relative mRNA levels) and release (protein expression levels) of three proinflammatory cytokines including IL-1β, IL-6 and TNF-α was studied. In M1 macrophages incubated on PLGA/Col and PLGA/Col NFs loaded with MET following the transition of macrophage phenotype to M2 type on NFs (Fig. 8). According to Fig. 8, compared with untreated cells, LPS/IFN γ stimulation considerably improved the levels of IL-1β, IL-6 and TNF-α in macrophages seeded on PLGA/Col NFs. After 3 days of macrophage culture on MET-PLGA/Col NFs, the expression level of IL 1β, IL-6 and TNF-α decreased significantly. This response further demonstrates that MET may successfully reduce IFNγ/LPS induced inflammation in macrophages.

Results from ELISA show that the highest levels of IL-1β, IL-6, and TNF-α production were found in the cells incubated on PLGA/Col NFs mats. As shown in Fig. 8, compared with pure NFs, it was observed that MET-PLGA/Col NFs could considerably decrease the protein concentration of mediastors of inflammation in LPS/IFN γ induced cells (≤ 0.01). The levels of IL-1β, IL-6, and TNF-α were also considerably greater in the LPS-activated macrophages imbedded on the MET-PLGA/Col NFs compared to the neat PLGA/Col demonstrating that the MET induce anti-inflammatory factors expression. According to our results, various in vivo and in vitro studies have revealed that MET could diminish the expression level of inflammatory cytokines and reduce the production of NO [64].

An appropriate wound healing medical device ought capable of reducing inflammation and promote cell proliferation. Thus, the progress of new connective tissue, re-epithelialization, and neovascularization are an important in order to address factors that contribute to nonphysiological remodeling and impaired tissue regeneration [65]. In a study, Le et al. designed the nanosized hydroxyapatite (HA) and showed that HA bioceramics noticeably ameliorate early inflammation by mediating macrophage polarization to the M2 phenotype, as well as reducing the secretion of inflammatory factors [66].

## Conclusion

In conclusion, we successfully manufactured smooth, uniform and bead-free MET-PLGA/Col NFs using electrospinning method. In vitro treatment of the macrophages with MET-PLGA/Col considerably upregulated M2 markers (Arg-1 and Fizz) and downregulated M1 markers (iNOS and SOCS3) expression levels in compared with PLGA/Col demonstrating the macrophage re-polarization from pro-inflammatory M1 to anti-inflammatory phenotype. Consequently, our research proposes an innovative therapeutic approach for treating inflammatory diseases by managing inflammation through handling of macrophage act using biomaterials, showing potential applications in the future.

## Data Availability

The datasets generated during and/or analyzed during the current study are available from the corresponding author (M.D.) on reasonable request.
